# 1-(3-Pyrid­yl)pyrrolidine-2,5-dione

**DOI:** 10.1107/S1600536809046522

**Published:** 2009-11-11

**Authors:** Hong-Bo Hou, Yi-Ming Liu, Luan-Fang Yang

**Affiliations:** a Department of Biology and Chemistry, Bao Shan College, BaoShan, Yun nan 678000, People’s Republic of China.

## Abstract

In the title mol­ecule, C_9_H_8_N_2_O_2_, the dihedral angle between the pyridine and the pyrrolidine rings is 64.58 (12)°. In the crystal structure, weak C—H⋯π-electron ring inter­actions stabilize the packing.

## Related literature

For general background to the pharmaceutical properties of pyrrolidine-2,5-dione derivatives, see: Procopiou *et al.* (1993 ▶); Obniska *et al.* (2009 ▶). 
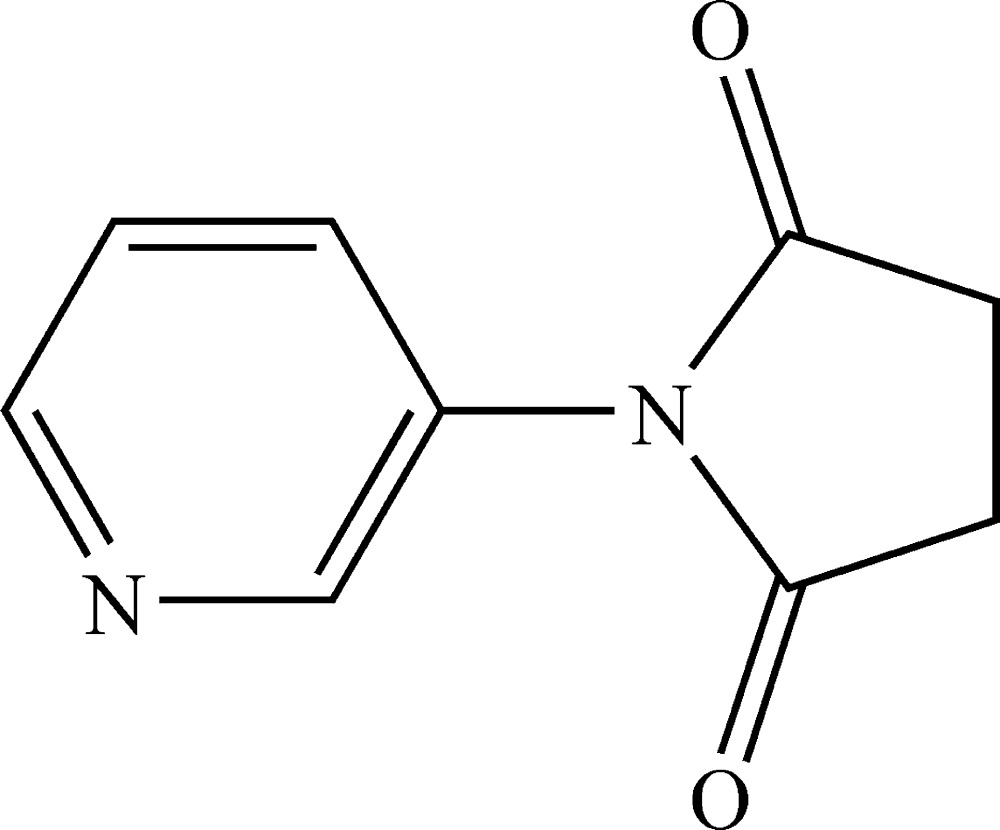


## Experimental

### 

#### Crystal data

C_9_H_8_N_2_O_2_
*M* *_r_* = 176.17
Orthorhombic, 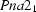

*a* = 12.137 (8) Å
*b* = 10.838 (6) Å
*c* = 6.831 (4) Å
*V* = 898.6 (9) Å^3^
*Z* = 4
Mo *K*α radiation
μ = 0.10 mm^−1^
*T* = 293 K
0.25 × 0.21 × 0.17 mm

#### Data collection

Bruker SMART CCD area-detector diffractometer
Absorption correction: multi-scan (*SADABS*; Bruker, 2005 ▶) *T*_min_ = 0.977, *T*_max_ = 0.984
3927 measured reflections
852 independent reflections
672 reflections with *I* > 2σ(*I*)
*R*_int_ = 0.073

#### Refinement

*R*[*F* ^2^ > 2σ(*F* ^2^)] = 0.033
*wR*(*F* ^2^) = 0.069
*S* = 1.00
852 reflections
119 parameters
1 restraint
H-atom parameters constrained
Δρ_max_ = 0.11 e Å^−3^
Δρ_min_ = −0.10 e Å^−3^

### 

Data collection: *SMART* (Bruker, 2002 ▶); cell refinement: *SAINT* (Bruker, 2002 ▶); data reduction: *SAINT*; program(s) used to solve structure: *SHELXS97* (Sheldrick, 2008 ▶); program(s) used to refine structure: *SHELXL97* (Sheldrick, 2008 ▶); molecular graphics: *ORTEP-3 for Windows* (Farrugia, 1997 ▶); software used to prepare material for publication: *WinGX* (Farrugia, 1999 ▶).

## Supplementary Material

Crystal structure: contains datablocks global, I. DOI: 10.1107/S1600536809046522/fb2172sup1.cif

Structure factors: contains datablocks I. DOI: 10.1107/S1600536809046522/fb2172Isup2.hkl

Additional supplementary materials:  crystallographic information; 3D view; checkCIF report

## Figures and Tables

**Table 1:** Hydrogen-bond geometry (Å)

*D*—H⋯*A*	*D*—H	H⋯*A*	*D*⋯*A*	*D*—H⋯*A*
C8—H8*B*⋯*Cg* ^i^	0.97	2.78	3.742 (6)	172

Symmetry code: (i) 
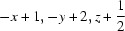
. *Cg* is the centroid of the N1,C1–C5 ring.

**Fig. 1.**
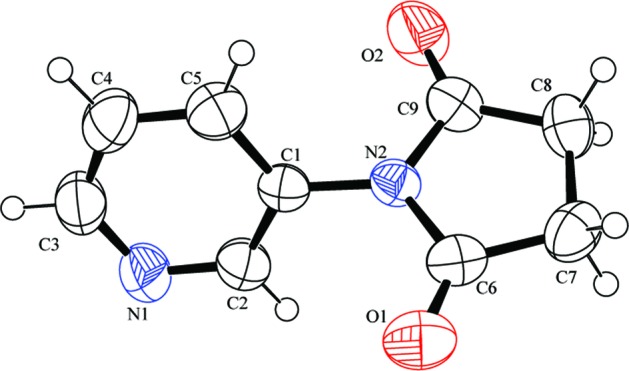
The title molecule with the atom-labelling scheme. The displacement ellipsoids are drawn at the 50% probability level.

## supplementary crystallographic information

### Comment

The derivatives of pyrrolidine-2,5-dione possess valuable pharmaceutical properties (Obniska *et al.*, 2009), among others are inhibitors of the cholesterol biosynthesis (Procopiou *et al.*, 1993). These interesting properties lead us to develop pyrrolidine derivatives containing the pyrrolidine-2,5-dione and the pyridine groups. In this paper, the synthesis of one of these compounds and its crystal structure are reported.

In the title molecule (Fig. 1), the dihedral angle between the pyridine and the pyrrolidine rings equals to 64.58 (12)°, There are C—H···π-electron ring interactions between the hydrogen atom H8B stemming from the pyrrolidine ring and the pyridine ring that serves as an acceptor (Tab. 1).

### Experimental

Solution of pyrrolidine-2,5-dione (0.04 mol) in ethanol (96%, 15 ml) was added to a stirred ethanol solution (96%, 25 ml) of 3-chloropyridine (0.04 mol) at room temperature, then KOH (0.01 mol) and tetrabutylammonium bromide (0.005 mol) was added to the resulting solution. This mixture was heated at 323 K for 4 h and then cooled to room temperature. After 30 ml of water had been added to this mixture, a white precipitate appeared. The mixture was filtered, the residue was dried under a reduced pressure in a vacuum drying box for 3 hours, then the residue was dissolved in 100 ml of ethanol (96%), and set aside for five days to obtain colourless block crystals suitable for X-ray analysis. Yield: 43%.

### Refinement

All the H atoms were discernible in the difference electron density maps. However, the hydrogens were constrained by the riding model approximation. *C*—H_methyl_=0.96 Å; *C*—H_aryl_=0.93 Å; *U*_iso_H_methyl_=1.5*U*_eq_(C_methyl_); *U*_iso_H_aryl_=1.2*U*_eq_(C_aryl_). In the absence of significant anomalous scattering effects 626 Friedel pairs have been merged.

### Crystal data

**Table:**

C_9_H_8_N_2_O_2_	*F*(000) = 368
*M**_r_* = 176.17	*D*_x_ = 1.302 Mg m^−^^3^
Orthorhombic, *P**n**a*2_1_	Mo *K*α radiation, λ = 0.71073 Å
Hall symbol: P 2c -2n	Cell parameters from 852 reflections
*a* = 12.137 (8) Å	θ = 2.5–25.0°
*b* = 10.838 (6) Å	µ = 0.10 mm^−^^1^
*c* = 6.831 (4) Å	*T* = 293 K
*V* = 898.6 (9) Å^3^	Block, colourless
*Z* = 4	0.25 × 0.21 × 0.17 mm

### Data collection

**Table:**

Bruker SMART CCD area-detector diffractometer	852 independent reflections
Radiation source: fine-focus sealed tube	672 reflections with *I* > 2σ(*I*)
graphite	*R*_int_ = 0.073
φ and ω scans	θ_max_ = 25.0°, θ_min_ = 2.5°
Absorption correction: multi-scan (*SADABS*; Bruker, 2005)	*h* = −13→14
*T*_min_ = 0.977, *T*_max_ = 0.984	*k* = −12→12
3927 measured reflections	*l* = −8→7

### Refinement

**Table:**

Refinement on *F*^2^	Secondary atom site location: difference Fourier map
Least-squares matrix: full	Hydrogen site location: difference Fourier map
*R*[*F*^2^ > 2σ(*F*^2^)] = 0.033	H-atom parameters constrained
*wR*(*F*^2^) = 0.069	*w* = 1/[σ^2^(*F*_o_^2^) + (0.0287*P*)^2^] where *P* = (*F*_o_^2^ + 2*F*_c_^2^)/3
*S* = 1.00	(Δ/σ)_max_ < 0.001
852 reflections	Δρ_max_ = 0.11 e Å^−^^3^
119 parameters	Δρ_min_ = −0.10 e Å^−^^3^
1 restraint	Extinction correction: *SHELXL97* (Sheldrick, 2008), Fc^*^=kFc[1+0.001xFc^2^λ^3^/sin(2θ)]^-1/4^
32 constraints	Extinction coefficient: 0.128 (8)
Primary atom site location: structure-invariant direct methods	

### Special details

**Table:**

Geometry. All e.s.d.'s (except the e.s.d. in the dihedral angle between two l.s. planes) are estimated using the full covariance matrix. The cell e.s.d.'s are taken into account individually in the estimation of e.s.d.'s in distances, angles and torsion angles; correlations between e.s.d.'s in cell parameters are only used when they are defined by crystal symmetry. An approximate (isotropic) treatment of cell e.s.d.'s is used for estimating e.s.d.'s involving l.s. planes.
Refinement. Refinement of *F*^2^ against ALL reflections. The weighted *R*-factor *wR* and goodness of fit *S* are based on *F*^2^, conventional *R*-factors *R* are based on *F*, with *F* set to zero for negative *F*^2^. The threshold expression of *F*^2^ > σ(*F*^2^) is used only for calculating *R*-factors(gt) *etc*. and is not relevant to the choice of reflections for refinement. *R*-factors based on *F*^2^ are statistically about twice as large as those based on *F*, and *R*- factors based on ALL data will be even larger.

### Fractional atomic coordinates and isotropic or equivalent isotropic displacement parameters (Å^2^)

**Table:**

	*x*	*y*	*z*	*U*_iso_*/*U*_eq_	
C1	0.46710 (19)	0.76727 (18)	0.7705 (4)	0.0421 (6)	
C2	0.5641 (2)	0.7232 (2)	0.8551 (4)	0.0576 (8)	
H2	0.5847	0.7545	0.9764	0.069*	
C3	0.5975 (2)	0.5948 (2)	0.5932 (5)	0.0653 (9)	
H3	0.6408	0.5352	0.5327	0.078*	
C4	0.5034 (2)	0.6353 (2)	0.4968 (4)	0.0661 (8)	
H4	0.4853	0.6040	0.3742	0.079*	
C5	0.4364 (2)	0.7235 (2)	0.5857 (5)	0.0563 (8)	
H5	0.3731	0.7523	0.5240	0.068*	
C6	0.34810 (18)	0.8409 (2)	1.0491 (4)	0.0508 (7)	
C7	0.29704 (19)	0.9616 (2)	1.1138 (4)	0.0578 (8)	
H7A	0.3299	0.9897	1.2353	0.069*	
H7B	0.2183	0.9524	1.1328	0.069*	
C8	0.3213 (2)	1.0532 (2)	0.9462 (5)	0.0565 (8)	
H8A	0.2534	1.0823	0.8874	0.068*	
H8B	0.3625	1.1237	0.9943	0.068*	
C9	0.38857 (18)	0.9809 (2)	0.8001 (5)	0.0505 (7)	
N1	0.62967 (18)	0.63755 (19)	0.7709 (4)	0.0668 (7)	
N2	0.40243 (14)	0.86060 (15)	0.8703 (3)	0.0417 (5)	
O1	0.34353 (16)	0.74092 (18)	1.1328 (4)	0.0784 (7)	
O2	0.42619 (16)	1.01652 (17)	0.6419 (4)	0.0797 (7)	

### Atomic displacement parameters (Å^2^)

**Table:**

	*U*^11^	*U*^22^	*U*^33^	*U*^12^	*U*^13^	*U*^23^
C1	0.0410 (13)	0.0403 (13)	0.0449 (17)	−0.0047 (10)	−0.0005 (13)	0.0029 (13)
C2	0.0566 (15)	0.0605 (16)	0.056 (2)	0.0107 (12)	−0.0101 (15)	−0.0018 (14)
C3	0.0721 (19)	0.0532 (16)	0.071 (2)	0.0082 (12)	0.0108 (19)	−0.0073 (16)
C4	0.0749 (18)	0.0639 (17)	0.060 (2)	−0.0014 (15)	−0.0085 (17)	−0.0176 (14)
C5	0.0496 (15)	0.0607 (17)	0.059 (2)	−0.0035 (12)	−0.0103 (14)	−0.0036 (15)
C6	0.0493 (14)	0.0581 (16)	0.0450 (19)	−0.0032 (13)	−0.0024 (13)	0.0052 (15)
C7	0.0484 (13)	0.0743 (17)	0.051 (2)	−0.0007 (13)	0.0042 (16)	−0.0066 (15)
C8	0.0445 (14)	0.0513 (15)	0.074 (2)	0.0018 (12)	0.0013 (14)	−0.0083 (16)
C9	0.0395 (12)	0.0501 (15)	0.062 (2)	−0.0033 (10)	0.0001 (14)	0.0065 (15)
N1	0.0668 (15)	0.0652 (15)	0.0684 (19)	0.0207 (11)	−0.0052 (15)	−0.0076 (15)
N2	0.0380 (10)	0.0427 (12)	0.0445 (14)	−0.0003 (8)	−0.0008 (10)	0.0044 (10)
O1	0.0992 (15)	0.0735 (13)	0.0624 (15)	0.0008 (11)	0.0115 (14)	0.0195 (11)
O2	0.0846 (13)	0.0681 (13)	0.0864 (17)	0.0147 (10)	0.0343 (13)	0.0302 (13)

### Geometric parameters (Å, °)

**Table:**

C1—C2	1.396 (4)	C6—O1	1.226 (3)
C1—C5	1.399 (4)	C6—N2	1.404 (3)
C1—N2	1.450 (3)	C6—C7	1.514 (4)
C2—N1	1.351 (3)	C7—C8	1.544 (4)
C2—H2	0.9300	C7—H7A	0.9700
C3—N1	1.356 (4)	C7—H7B	0.9700
C3—C4	1.390 (4)	C8—C9	1.509 (4)
C3—H3	0.9300	C8—H8A	0.9700
C4—C5	1.393 (4)	C8—H8B	0.9700
C4—H4	0.9300	C9—O2	1.235 (4)
C5—H5	0.9300	C9—N2	1.400 (3)
			
C2—C1—C5	118.8 (2)	C6—C7—H7A	110.7
C2—C1—N2	120.0 (3)	C8—C7—H7A	110.7
C5—C1—N2	121.1 (2)	C6—C7—H7B	110.7
N1—C2—C1	123.8 (3)	C8—C7—H7B	110.7
N1—C2—H2	118.1	H7A—C7—H7B	108.8
C1—C2—H2	118.1	C9—C8—C7	105.1 (2)
N1—C3—C4	123.6 (3)	C9—C8—H8A	110.7
N1—C3—H3	118.2	C7—C8—H8A	110.7
C4—C3—H3	118.2	C9—C8—H8B	110.7
C3—C4—C5	119.3 (3)	C7—C8—H8B	110.7
C3—C4—H4	120.4	H8A—C8—H8B	108.8
C5—C4—H4	120.4	O2—C9—N2	123.1 (2)
C4—C5—C1	118.1 (3)	O2—C9—C8	128.1 (2)
C4—C5—H5	121.0	N2—C9—C8	108.8 (3)
C1—C5—H5	121.0	C2—N1—C3	116.5 (2)
O1—C6—N2	124.2 (2)	C9—N2—C6	112.6 (2)
O1—C6—C7	127.5 (3)	C9—N2—C1	123.6 (2)
N2—C6—C7	108.3 (2)	C6—N2—C1	123.8 (2)
C6—C7—C8	105.1 (2)		

### Hydrogen-bond geometry (Å, °)

**Table:**

*D*—H···*A*	*D*—H	H···*A*	*D*···*A*	*D*—H···*A*
C8—H8B···Cg^i^	0.97	2.78	3.742 (6)	172

Symmetry codes: (i) −*x*+1, −*y*+2, *z*+1/2.
